# Cell death in amastigote forms of *Leishmania amazonensis* induced by parthenolide

**DOI:** 10.1186/1471-2180-14-152

**Published:** 2014-06-10

**Authors:** Tatiana Shioji Tiuman, Tânia Ueda-Nakamura, Antonio Alonso, Celso Vataru Nakamura

**Affiliations:** 1 Programa de Pós-Graduação em Ciências Farmacêuticas, Universidade Estadual de Maringá, Av. Colombo 5790, 87020-900 Maringá, Paraná, Brazil; 2 Instituto de Física, Universidade Federal de Goiás, Campus II, CEP 74001-970 Goiânia, Goiás, Brazil

**Keywords:** *Leishmania amazonensis*, Amastigotes, Parthenolide, Cell death, Autophagy

## Abstract

**Background:**

*Leishmania amazonensis* infection results in diverse clinical manifestations: cutaneous, mucocutaneous or visceral leishmaniasis. The arsenal of drugs available for treating *Leishmania* infections is limited. Therefore, new, effective, and less toxic leishmaniasis treatments are still needed. We verified cell death in amastigote forms of *Leishmania amazonensis* induced by the sesquiterpene lactone parthenolide.

**Results:**

The tested compound was able to concentration-dependently affect axenic and intracellular amastigotes, with IC_50_ values of 1.3 μM and 2.9 μM, respectively after 72 h incubation. No genotoxic effects were observed in a micronucleus test in mice. Parthenolide induced morphological and ultrastructural changes in axenic amastigotes, including a loss of membrane integrity, swelling of the mitochondrion, cytoplasmic vacuoles, and intense exocytic activity in the region of the flagellar pocket. These results led us to investigate the occurrence of autophagic vacuoles with monodansylcadaverine and the integrity of the plasma membrane and mitochondrial membrane potential using flow cytometry. In all of the tests, parthenolide had positive results.

**Conclusions:**

Our results indicate that the antileishmanial action of parthenolide is associated with autophagic vacuole appearance, a reduction of fluidity, a loss of membrane integrity, and mitochondrial dysfunction. Considering the limited repertoire of existing antileishmanial compounds, the products derived from medicinal plants has been one the greatest advances to help develop new chemotherapeutic approaches.

## Background

Leishmaniasis is associated with high morbidity but low mortality. It is a poverty-related disease and has become a serious impediment to socioeconomic development. The true burden of this illness remains unclear because the notification of the disease is compulsory in only 32 of the 88 affected countries, and most of the affected people live in remote areas. Additionally, the disfiguring scars caused by *Leishmania* keep patients hidden. An estimated 1.5 million new cases of cutaneous leishmaniasis and 500,000 cases of visceral leishmaniasis occur annually, with approximately 12 million people currently infected [1]. Moreover, cases of *Leishmania* and human immunodeficiency virus co-infection are becoming more frequent [2,3].

*Leishmania* (*Leishmania*) *amazonensis* infection results in diverse clinical manifestations, ranging from cutaneous to mucocutaneous or visceral involvement [4]. This is attributable to the genetic diversity of *L. amazonensis* strains, and this divergence extends to variations of chromosome size [5].

The arsenal of drugs available for treating *Leishmania* infections is limited. The basic treatment consists of administering pentavalent antimonial compounds [6]. However, the choice of medication depends on the species involved and type of clinical manifestation [7]. The usefulness of antileishmanial drugs has been limited by their toxicity, and treatment failure is often attributable to drug resistance [8]. To solve this problem, developing less toxic drugs and discovering cellular and molecular markers in parasites to identify the phenotype of chemoresistance against leishmanicidal drugs are necessary [8,9]. These problems led to the development of additional antileishmanial drugs. Some drug-delivery systems, plants, and synthetic compounds are being developed as effective treatments for the disease [7].

Previous studies demonstrated the *in vitro* activity of parthenolide, a sesquiterpene lactone purified from *Tanacetum parthenium*, against promastigotes and intracellular amastigotes (inside J774G8 macrophages) of *L. amazonensis*[10]. Moreover, significant alterations in promastigote forms were demonstrated by light microscopy and scanning and transmission electron microscopy [11].

We evaluated the activity of parthenolide against *L. amazonensis* axenic amastigotes and demonstrated a possible mechanism of action of this compound in this life stage of the parasite.

## Results

### Antileishmanial assays

**Figure 1:**
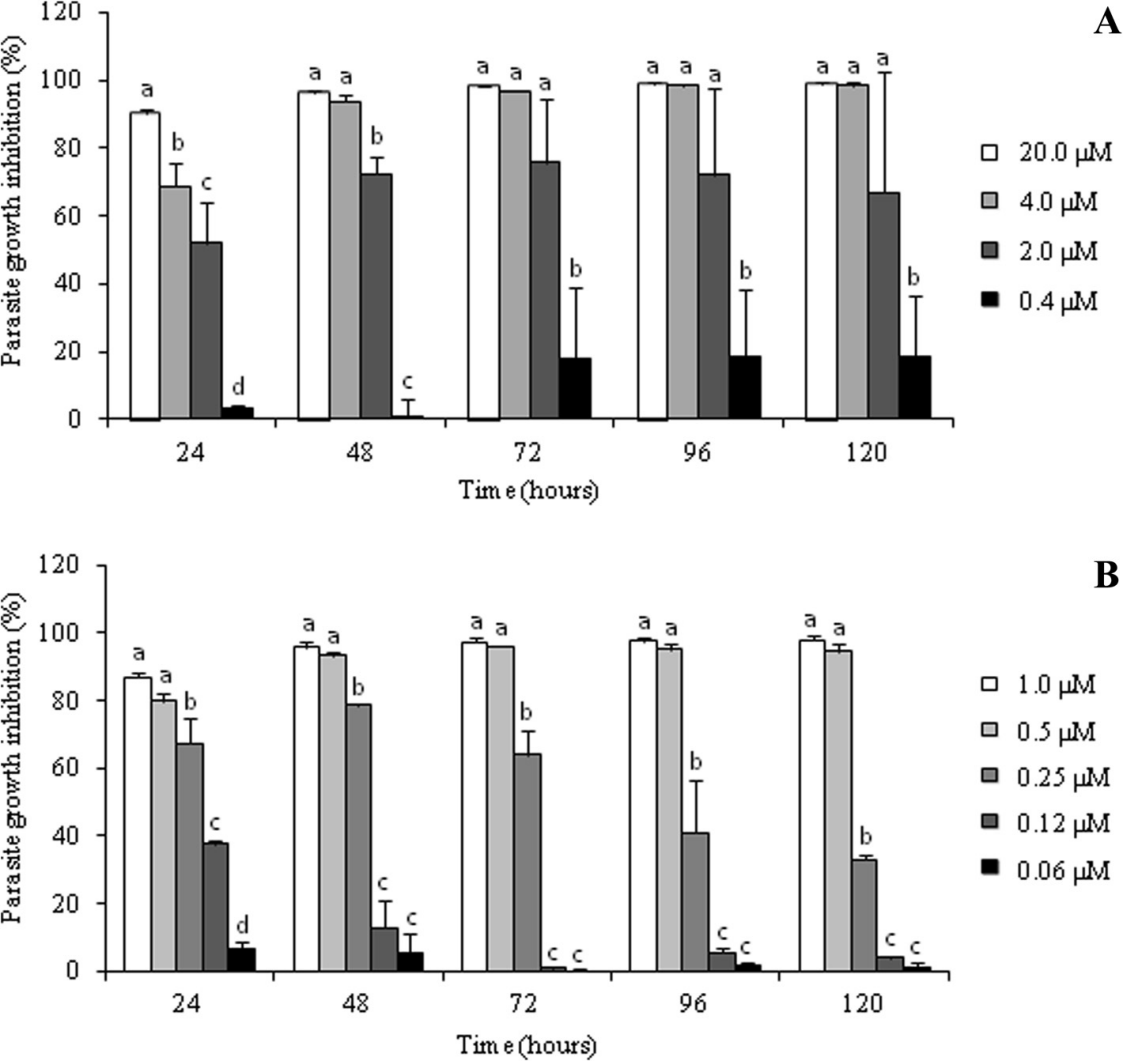
**Effects of parthenolide (A) and amphotericin B (B) on the growth of *L. amazonensis *axenic amastigotes.** After treatment with different concentrations of the drugs, parasites were counted, and the percentage of parasite growth inhibition was determined daily for 120 h. The data indicate the average of the two independent experiments performed twice. Statistical analysis: the data of each incubation period were compared statistically at p < 0.05. Bars that are not indicated with letters in common are statistically different.

The addition of 4.0 μM parthenolide to the culture of axenic amastigotes induced growth arrest and partial cell lysis after 48 h (i.e., growth inhibition up to 90%). When the cells were treated with 2.0 μM parthenolide, the percentage of growth inhibition was approximately 70%. Parthenolide had an IC_50_ of 1.3 μM and IC_90_ of 3.3 μM after 72 h incubation (Figure 1A).

A concentration of 1.0 or 0.5 μM of the reference drug amphotericin B inhibited more than 93% of *L. amazonensis* amastigote cell growth. This drug had an IC_50_ and IC_90_ of 0.22 μM and 0.45 μM, respectively, after culturing for 72 h (Figure 1B).

**Figure 2:**
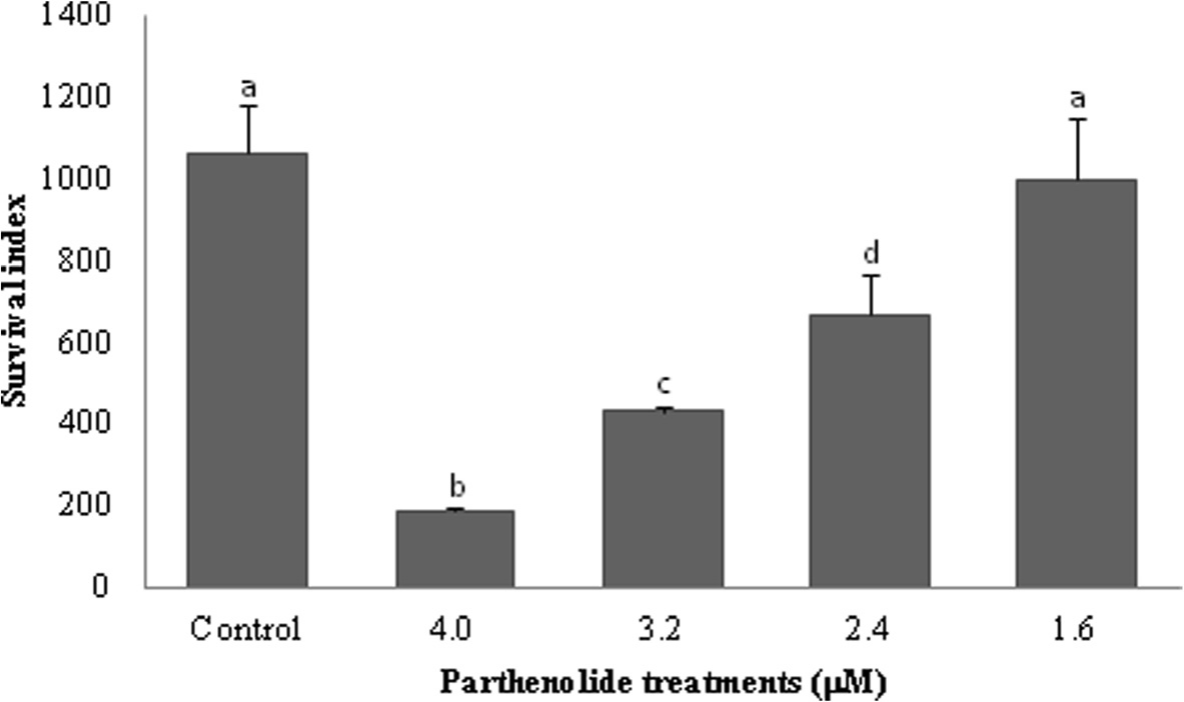
**Effect of parthenolide on amastigotes of *L. amazonensis *in mouse resident peritoneal macrophages.** Peritoneal macrophage cells were infected with promastigote forms, and then intracellular amastigotes were treated with different concentrations of parthenolide. After 24 h treatment, the survival index was calculated by multiplying the percentage of macrophages with internalized parasites and mean number of internalized parasites per macrophage. The results shown are from one representative experiment of two independent experiments performed in duplicate. The data were compared statistically at p < 0.05. Bars that are not indicated with letters in common are statistically different.

Parthenolide also inhibited the growth of intracellular amastigotes in mouse resident peritoneal macrophages after 24 h incubation. Treatment with 4.0, 3.2, 2.4, and 1.6 μM parthenolide reduced the proliferation of parasites into macrophages (survival index) by 82.5, 59.4, 37.3, and 6.1%, respectively, compared with the control. The survival index indicated that parthenolide inhibited the intracellular viability and multiplication of *Leishmania* in infected murine macrophages and showed 50% inhibition of cell survival at a concentration of 2.9 μM (Figure 2).

Previous studies showed that when J774G8 murine macrophages were treated with parthenolide, the 50% cytotoxic concentration (CC_50_) was 56.4 μM [10]. By comparing the toxicity for J774G8 macrophages and activity against intracellular amastigotes, obtaining the selectivity index ratio is possible (CC_50_ for J774G8 cells/IC_50_ for protozoa). In the present study, parthenolide had an IC_50_ of 2.9 μM, presenting a selectivity index ratio of 19.4 (i.e., the compound is 19.4-times more selective against parasites than host cells).

### Mutagenicity evaluation

The results of the *in vivo* bone marrow micronucleus test in rats are shown in Table 1. Parthenolide did not induce genotoxic effects at a concentration of 3.75 mg/kg body weight, with no significant increase in the frequency of MNPCE (10.0 ± 1.6) compared with the vehicle control group (7.0 ± 1.8). In contrast, a significant increase in the frequency of MNPCE was observed in the positive control group (cyclophosphamide; 27.0 ± 4.0). In the present study, no clinical signs of toxicity were observed in treated animals. However, further studies should be performed with higher concentrations of parthenolide to exclude the possibility of genotoxicity.

**Table 1:** Micronucleated polychromatic erythrocyte (MNPCE) score in 2,000 reticulocytes from bone marrow of mice

**Treatment**	**MNPCE (mean ± SD)**
Vehicle	7.0 ± 1.8
Cyclophosphamide	27.0 ± 4.0^b^
Parthenolide	10.0 ± 1.6^a^

Findings after 24 h oral treatment with vehicle (negative control), cyclophosphamide (positive control), and parthenolide.

^a^No significant difference compared with negative control and significant difference compared with positive control (*p* < 0.05).

^b^Significant difference compared with negative control (*p* < 0.05).

### Scanning and transmission electron microscopy

**Figure 3:**
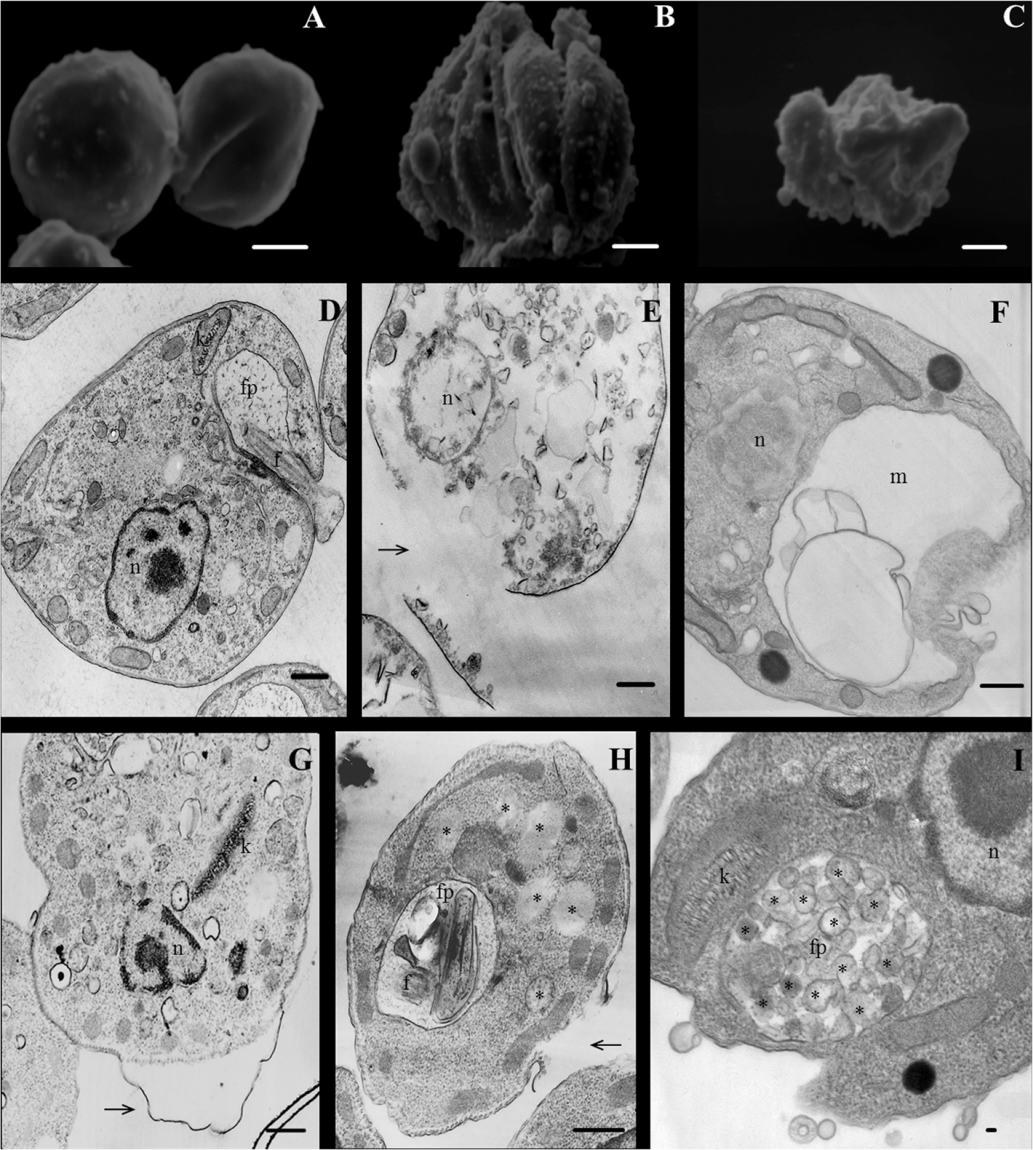
**Scanning (A-C) and transmission (D-I) electron microscopy of axenic amastigotes of *L. amazonensis *treated with parthenolide.** Amastigotes were incubated for 72 h in the absence **(A and D)** or presence **(B, C, F-I)** of the IC_50_ (1.3 μM) of parthenolide. For transmission electron microscopy, the treatment of amastigotes was also accomplished using the IC_50_ of amphotericin B as a reference drug that acts on the cytoplasmic membrane **(E)**. The arrows indicate plasma membrane blebs or loss of membrane integrity, and the asterisks indicate vesicles located in the cytoplasm or flagellar pocket. n, nucleus; f, flagellum; fp, flagellar pocket; m, mitochondrion; k, kinetoplast. Scale bars **=** 1 μm.

To determine the morphological and ultrastructural changes in *L. amazonensis* axenic amastigotes induced by parthenolide, the cells were treated with the IC_50_ (1.3 μM) of the compound. Untreated controls showed no morphological (Figure 3A) or ultrastructural (Figure 3D) differences. Similarly, cells incubated with 0.05% DMSO (i.e., the same concentration used in the final solutions of parthenolide) remained unaltered (data not shown). When treated with parthenolide, changes in form were visualized by scanning electron microscopy (Figure 3B and C). Transmission electron microscopy showed a loss of membrane integrity associated with amphotericin B exposure at the IC_50_ concentration (Figure 3E). Parthenolide caused intense swelling of the mitochondrion (Figure 3F) and cytoplasmic blebbing (Figure 3G). Finally, the ultrastructural analysis showed that amastigotes treated with parthenolide formed multiple cytoplasmic vacuoles (Figure 3H), and intense exocytic activity was observed in the region of the flagellar pocket, appearing as concentric membranes within the pocket (Figure 3I).

### Labeling of autophagic vacuoles with monodansylcadaverine

**Figure 4:**
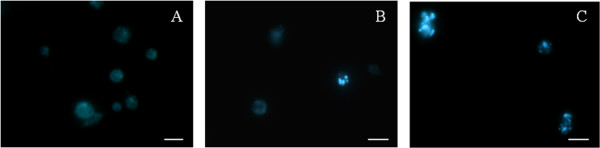
**Monodansylcadaverine (MDC)-labeled vesicles in axenic amastigotes of *L. amazonensis *induced by parthenolide treatment.** Amastigotes were incubated for 72 h in Schneider medium (control cells) **(A)** or in the presence of the IC_50_**(B)** or IC_90_**(C)** of parthenolide. The data are representative of at least three independent experiments. Scale bars = 5 μm.

We studied the incorporation of monodancylcadaverine (MDC) in cells in which autophagy was stimulated by parthenolide. Axenic amastigotes treated with the IC_50_ (Figure 4B) or IC_90_ (Figure 4C) of parthenolide showed an increase in the number of vesicles, indicating that the compound induced the formation of MDC-labeled vacuoles in the cytoplasm. MDC-positive cells were visualized in treated cells but not in control cells (Figure 4A) or amphotericin-treated cells (data not shown). These results show that parthenolide treatment, unlike amphotericin B, led to the formation of autophagic vacuoles in *L. amazonensis* amastigotes.

### Flow cytometric measurement of amastigote culture

**Figure 5:**
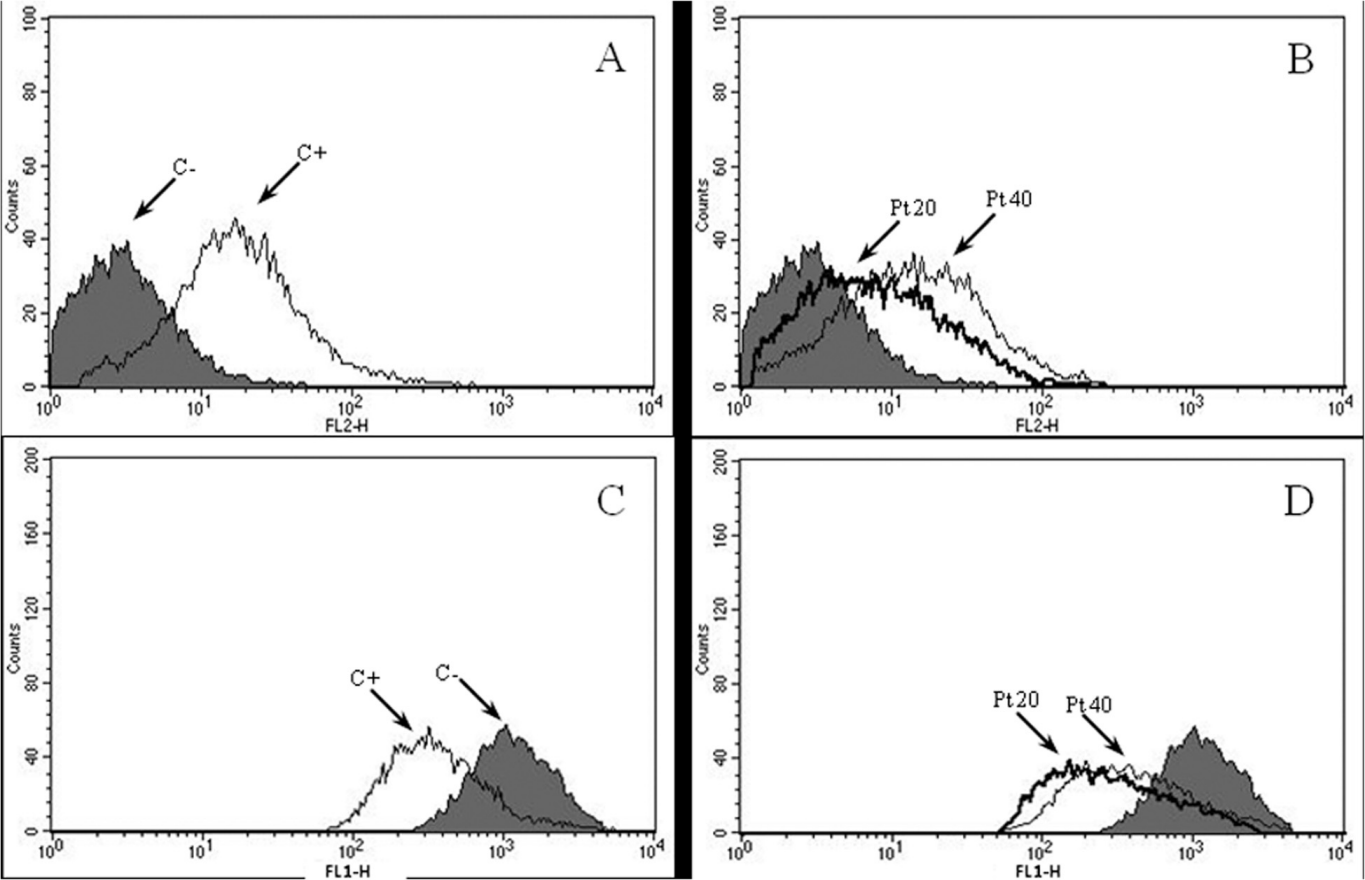
**Flow cytometry analysis of propidium iodide- (A, B) and rhodamine 123- (C, D) labeled axenic amastigotes of *L. amazonensis*. (A)** Untreated cells: negative control (C-) and amphotericin B as positive control (C+). **(B)** Amastigotes treated with 20 or 40 μM parthenolide (Pt 20 or Pt 40). **(C)** Untreated cells: negative control and carbonyl cyanide m-chlorophenylhydrazone as a positive control. **(D)** Amastigotes treated with 20 or 40 μM parthenolide (Pt 20 or Pt 40). The data are representative of at least two independent experiments.

Live *L. amazonensis* cells were incubated with propidium iodide and rhodamine 123, and fluorescence was measured by flow cytometry. The gated percentage of propidium iodide-stained amastigotes after treatment with amphotericin B (positive control) was 71.4%, much higher than untreated parasites (negative control) that presented 6.0% (Figure 5A). When the cells were treated with 20 and 40 μM parthenolide, the percentages of labeled amastigotes were 34.2% and 56.2%, respectively (Figure 5B), possibly indicating a considerable increase in plasma membrane permeability. To prove that *Leishmania* cells functionally respond to the pharmacological alteration of ΔΨ_m_, amastigotes were treated with the protonophore carbonyl cyanide *m*-chlorophenylhydrazone (CCCP), which has been shown to interfere with mitochondrial membrane potential in various cell types [12]. The results showed that 82.5% of the amastigotes without treatment (negative control) presented a maximal increase in fluorescence, and with 200 μM CCCP, 46.7% showed fluorescence, indicating a loss of ΔΨ_m_ (Figure 5C). We next observed ΔΨ_m_ reductions of 68.4% and 56.1% when the amastigotes were treated with 20 and 40 μM parthenolide, respectively, suggesting that this compound interferes with the mitochondrial membrane potential leading to alteration of ATP generation and in consequence cell damage takes place.

### EPR spectra of spin-labeled Leishmania

**Figure 6:**
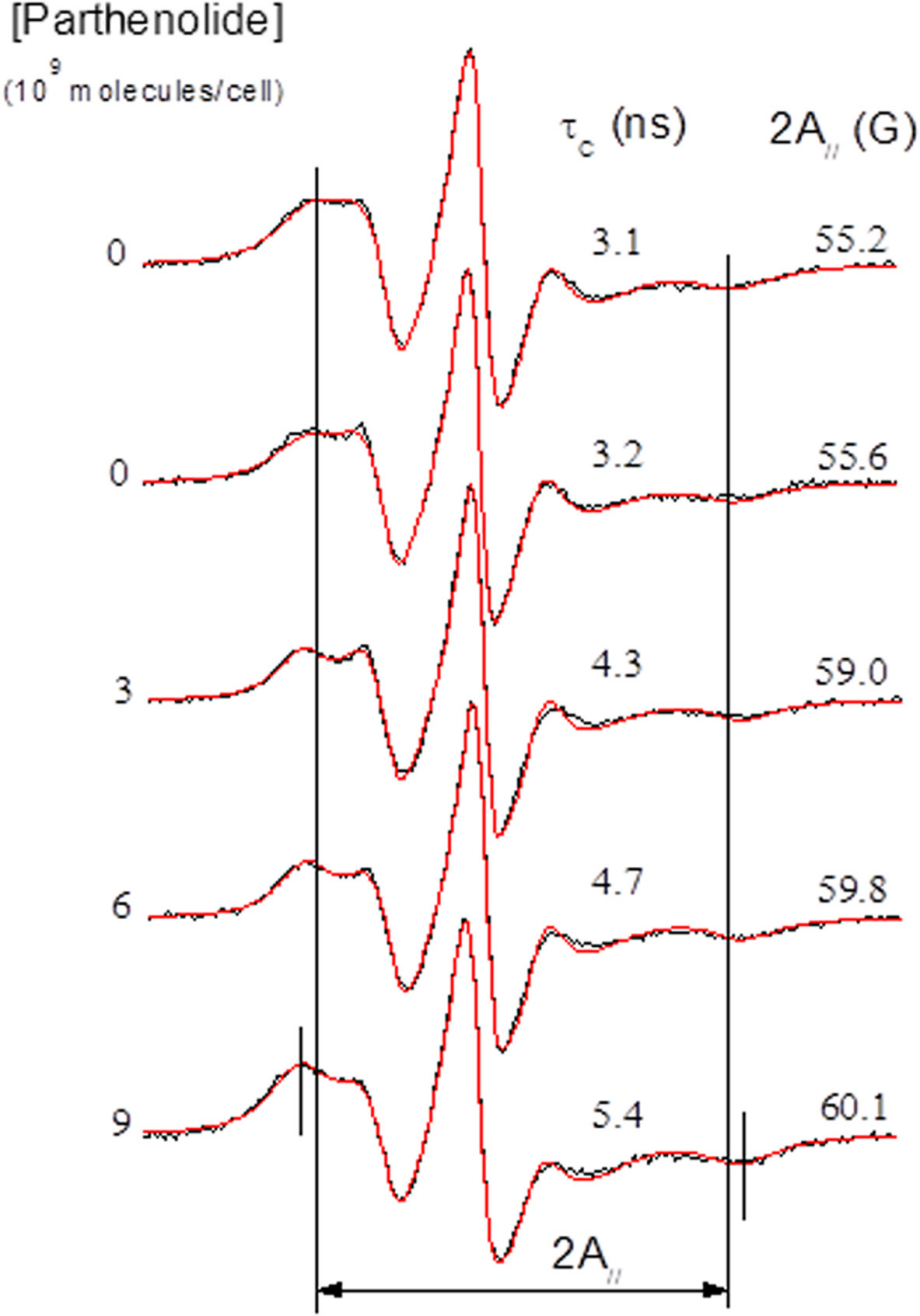
**Experimental EPR spectra (black line) and theoretical fits (red line) of spin-label 5-DSA in *Leishmania *membrane.** The experiment was conducted at 26°C for samples untreated and treated with parthenolide at the indicated concentrations. EPR spectra were simulated with the NLLS fitting program, and the values of the parameter rotational correlation time, *τ*_*C*_, obtained from the fit for each spectrum are indicated on a nanosecond scale. The EPR parameter 2A_//_is the separation in magnetic-field units between the first and last resonance lines of the spectrum. The vertical lines indicate the 2A_//_for the control samples, and the smaller vertical lines illustrate the increase in 2A_//_for the sample treated with 9 × 10^9^ molecules/cell. The measured 2A_//_values and τ_C_ values indicate that the presence of parthenolide significantly reduced lipid fluidity. The estimated experimental errors for the 2A_//_and τ_C_ parameters are 0.5 G and 1.0 ns, respectively.

The experimental and best-fit EPR spectra of spin-label 5-DSA structured in the plasma membrane of *Leishmania* are shown in Figure 6. These EPR spectra are typical for cellular membranes that contain an appreciable amount of integral proteins. Treatment with parthenolide increased two EPR parameters, the outer hyperfine splitting, 2A_//_, and rotational correlation time, *τ*_*C*_, indicating a significant reduction of membrane lipid dynamics. 2A_//_is a practice parameter measured directly in EPR spectra that has been widely used to monitor membrane fluidity, although in principle it is a static parameter associated with the orientation distribution of the spin labels in the membrane. The theoretical EPR spectrum of spin-label 5-DSA in the plasma membrane of *Leishmania* was best fitted using a model of two spectral components. This indicates that the membrane has two populations of spin labels of distinct mobility. The EPR spectra of spin labels in lipid bilayers are well known to contain proteins sometimes composed of two spectral components. The more restricted component is associated with boundary lipids where the spin labels surround the hydrophobic regions of proteins, whereas the more mobile component arises from the spin labels located in the bulk bilayer phase, away from the protein [13]. The fitting program provides the *τ*_*c*_ and population of each component. Thus, the mean of the rotational correlation time was calculated as *τ*_*c*_ *= N*_1_*τ_*c1*_ *+ N*_2_*τ_*c2*_, in which *N*_*1*_ and *N*_*2*_ are the fractions of the population in components 1 and 2, respectively, and *τ*_*c1*_ and *τ*_*c2*_ are the corresponding rotational time correlations.

## Discussion

For many years, parasites of the genus *Leishmania* have displayed extraordinary plasticity to face modifications in their environment [14]. The expansion of risk factors related to environmental changes and man-made transformations are making leishmaniasis a growing public health concern in many countries worldwide [15]. Leishmaniasis urgently needs novel drugs with improved features, and many compounds primarily derived from plants are promising leads for the development of novel chemotherapeutics [16].

The development of axenic cultures of amastigotes of *Leishmania* species yielded new opportunities to investigate the antileishmanial activities of new compounds directly at the mammalian stage of the parasite [17]. Assays that use intracellular amastigote cell cultures are relevant because this life cycle stage of the parasite is important to its pathogenicity, and data obtained exclusively from promastigote cell lines are insufficient [16]. Therefore, in the present study, we determined the leishmanicidal activity of parthenolide, which is naturally occurring, in both axenic and intracellular amastigotes.

To discover better leishmanicidal compounds, the isolation and purification of the active ingredients of medicinal plants are gaining attention [18]. Many new natural product groups, such as terpenes, have exhibited antiprotozoal potential and attracted renewed interest with surprising efficacy and selectivity [19].

Parthenolide is a lipophilic hydrocarbon compound formed by units of isoprene. The accumulation of lipophilic compounds in the cytoplasmic membrane and membrane constituents of microorganisms has considerable effects on the loss of cellular integrity and inhibition of respiratory cellular activity in mitochondria [20]. This interaction with cell membranes eventually leads to cell death. In our research, parthenolide had antileishmanial effects against axenic and intracellular amastigotes of *L. amazonensis* presenting IC_50_ of 1.3 after 72 h growth and 2.9 μM after 24 h growth, respectively. The differences in IC_50_ values can be explained because the experiments with axenic amastigotes are directed against the relevant stage of the parasite whereas the use of intracellular amastigotes will give essential information on the capacity of the drugs to target intracellular organisms. The role played by the macrophages on drug-mediated toxicity may be important. Their presence may limit the availability of the compounds under evaluation [21,22].

The toxicity for J774G8 macrophages and the activity against intracellular amastigotes were compared by using the selectivity index ratio (CC_50_ for J774G8 cells/IC_50_ for protozoa) [10]. The parthenolide was more selective against the intracellular amastigotes than the mammalian cells, with a selectivity index ratio of 19.4. It is generally considered that biological efficacy is not due to *in vitro* cytotoxicity when this index is ≥ 10 [23,24].

The low toxicity against mammalian cells is an important criterion in the search for active compounds with antiprotozoal activity. For this purpose, the genotoxicity of parthenolide in a mouse model was determined using a micronucleus test and cyclophosphamide as the positive control because it is a known genotoxin [25]. Micronuclei are masses of cytoplasmic chromatin that appear outside the main nucleus as a result of chromosomal damage or damage to the mitotic apparatus in the erythroblasts of the test species, and they can be used as an indicator of the effects of agents that cause DNA damage [26]. In mice, micronuclei in mature erythrocytes in peripheral blood live approximately 1 month, providing a measure of average chromosomal damage [27]. Our results showed no differences in the frequency of MNPCE compared with the negative control, demonstrating no toxic effects on bone marrow at the dose tested (3.75 mg/kg body weight).

Electron microscopic studies revealed extensive cytoplasmic vacuolization, leading to the examination of the possibility that parthenolide induces autophagic cell death. Autophagy cell death is a process that is thought to occur in all eukaryotes and is characterized by an accumulation of autophagic vacuoles. This mechanism occurs for energy production for survival when cells recycle their cytoplasmic contents during periods of environmental stress or certain stages of development. A double-membrane vesicle called the autophagosome forms in the cytosol, engulfing organelles and bulk cytoplasm. Subsequently, these vesicles fuse with lysosomes, where their contents are degraded and recycled [28]. One of the most frequently used methods to examine autophagy is staining with acidotropic dyes [29], and MDC is considered an autofluorescent compound and specific marker for autophagic vacuoles [30]. MDC staining is only obtained when the compartments into which it loads are acidic. Neutralization of these compartments leads to a swift loss of MDC staining or lack of MDC uptake [31]. Therefore, we suggest that the vacuoles that were observed under a transmission electron microscope are autophagosomes. Another study used MDC as a marker to analyze the molecular level of the machinery involved in the autophagic process [32] and was also used to demonstrate that antimicrobial peptides induce autophagic cell death in *L. donovani*[33].

Amphotericin B was used as a positive control in some of our experiments because this polyene antibiotic forms aqueous and nonaqueous pores in membranes, which is the basis of leishmanicidal action [34]. Using transmission electron microscopy, we could see the loss of membrane integrity induced by this antimicrobial agent. Similarly, alterations in the cytoplasmic membrane, including membrane blebbing and disruption, could be visualized in axenic amastigotes treated with parthenolide. Studies have shown that a flow cytometric membrane potential assay can be used as a reliable tool for studying the interactions between amphotericin B and the *Leishmania* membrane [35]. Alterations in membrane permeability are detected by propidium iodide nucleic acid stain that selectively passes through plasma membranes and bind to DNA, emitting high fluorescence when excited by an argon ion laser [36]. Since its introduction, the propidium iodide flow cytometric assay has also been widely used as a quantitative measure of cell apoptosis. During apoptosis, DNA fragmentation occurs, with a subsequent loss of cellular DNA content [37].

Terpenoic compounds can produce major changes in the cellular and mitochondrial membrane structures of different pathogenic agents, modifying their permeability and integrity [20]. Ultrastructural findings also revealed mitochondrial damage induced by parthenolide. We used flow cytometry analysis to determine whether the compound interferes with the mitochondrial membrane potential of the amastigotes. The flow cytometry results showed that transmembrane potential decreased, reflected by a reduction of rhodamine 123 fluorescence. Rhodamine 123 is a fluorescent cationic stain for mitochondria in living cells and is subsequently washed out of the cells once the mitochondrion’s membrane potential is lost [38]. The present results indicated an increase in proton permeability through the internal mitochondrial membrane, inhibition of electron transport, or decrease in mitochondrial substrate transport/oxidation, which would impair proton pumping by mitochondrial complexes and reduce adenosine triphosphate synthesis, resulting in parasite cell death [39]. CCCP was used as positive control because it is an uncoupler of oxidative phosphorylation and reduces mitochondrial membrane potential by directly attacking the proton gradient across the inner mitochondrial membrane [12,40]. Amastigotes treated with parthenolide presented severe plasma membrane and mitochondrial damage, suggesting an autophagic process [39].

Treatment with parthenolide induced shedding of the membranes into the flagellar pocket, appearing as concentric membranes and suggesting intense exocytic activity because this site is where endocytosis and exocytosis occur in trypanosomatids. Treatment of promastigote forms of *L. amazonensis* with edelfosine for 1 day [41] and parthenolide for 3 days [10] also led to the appearance of a large number of vesicles inside the flagellar pocket, suggesting a process of exacerbated protein production by cells as they attempt to survive.

Other studies indicated that the plasma membrane of human promyelocytic leukemic HL-60 cells appears to be one of the targets of parthenolide because its integrity is lost very early during cell death, reflected by atypical apoptosis and primary necrosis (i.e., lysis of the membrane) [42].

The lipid spin probe 5-DSA was incorporated into the plasmatic membrane of *Leishmania* in the usual way, and the EPR spectra obtained were typical for cell membranes. Interestingly, the spectra of the *Leishmania* membrane were very similar to those for the same spin label in erythrocyte membranes [43]. The erythrocyte membrane of spin-labeled lipids has been well characterized by EPR spectroscopy and is considered to have certain rigidity, particularly because of its high content of protein and cholesterol. The presence of sesquiterpene parthenolide significantly increased the rigidity of the membrane of *Leishmania* when applied to the cell suspension at a ratio of 3 × 10^9^ parthenolide molecules/cell. Parthenolide also showed dose-dependent anti-*Leishmania* activity against the amastigote form. The IC_50_ was 1.3 μM parthenolide/ml for a cell concentration of 1 × 10^6^ cell/ml. Therefore, the effect of parthenolide against the amastigote forms of *Leishmania* was observed at a ratio of 7.8 × 10^8^ parthenolide molecules/cell. The greatest change in membrane fluidity was observed at a concentration 3.8-fold higher than for growth inhibition. Membrane stiffness, assessed by EPR spectroscopy of the spin label, has been associated with lipid peroxidation [44,45]. A detailed study of the interaction between parthenolide and membranes and their role as a pro-oxidant in simpler systems is necessary to determine whether the membrane rigidity observed here was attributable to lipid peroxidation.

## Conclusions

Our results indicated that the antileishmanial action of parthenolide is associated with autophagic vacuole appearance, membrane stiffness, the loss of membrane integrity, and mitochondrial dysfunction. These results indicate that parthenolide induced amastigote cell death by autophagy, but other mechanisms of cell death cannot be dismissed, such as apoptosis and necrosis. Considering the limited repertoire of existing antileishmanial compounds, continuously developing new leishmanicidal compounds is essential. In the ongoing search for the best antileishmanial compounds, products derived from plants are gaining ground. The isolation and purification of the active components of medicinal plants has been one the greatest advances. Additionally, delineation of the biochemical mechanisms involved in mediating effect of these compounds would help develop new chemotherapeutic approaches.

## Methods

### Drugs

Parthenolide (minimum 90%) was purchased from Sigma-Aldrich (Steinheim, Germany). Amphotericin B (Cristália, Produtos Químicos Farmacêuticos Ltda, Itapira, SP, Brazil) was used as a positive control. In all of the tests, 0.05% dimethyl sulfoxide (DMSO; Sigma, St. Louis, MO, USA) was used to dissolve the highest dose of the compounds and had no effect on the parasites’ proliferation or morphology.

### Axenic amastigotes

Promastigotes of the *Leishmania* species differentiate to amastigotes with the combination of low pH and high temperature [46]. The WHOM/BR/75/Josefa strain of *Leishmania amazonensis*, isolated by C.A. Cuba-Cuba (University of Brasília, Brasília, Distrito Federal, Brazil) from a human case of diffuse cutaneous leishmaniasis, was used in the present study. Axenic amastigote cultures were obtained by the *in vitro* differentiation of promastigotes from the stationary phase in 25 cm^2^ tissue culture flasks by progressive temperature increase and pH decrease [47]. The cultures were maintained at 32°C in Schneider’s insect medium (Sigma, St. Louis, MO, USA), pH 4.6, with 20% fetal bovine serum through weekly serial sub-culturing for further studies.

### Antiproliferative effect

For the parasite growth inhibition assays, *L. amazonensis* axenic amastigotes were harvested during the exponential phase of growth, and 10^6^ cells were added to each well of a 24-well plate and treated with different concentrations of parthenolide and amphotericin B. Medium alone and 0.05% DMSO were used as negative controls. For each treatment, the parasites were observed and counted daily using a Neubauer chamber with an optical microscope. Each experiment was performed in duplicate and twice on different occasions. The antiproliferative effect (percentage of growth inhibition) was evaluated with 5 day treatment, and the data are expressed as the mean ± standard error of the mean (Microsoft Excel). The corresponding 50% and 90% inhibitory concentrations (IC_50_ and IC_90_) were determined from the concentration-response curves (Excel software). Data were compared via one-way analysis of variance (ANOVA) followed by Tukey’s multiple range test for statistically significant differences at p < 0.05.

### Activity of parthenolide in infection of murine macrophages

The effect of parthenolide on *L. amazonensis*-infected mouse peritoneal macrophages was evaluated. The experimental protocol was approved by the Animal Ethics Committee of the Universidade Estadual de Maringá (no. 013/2010). BALB/c mice resident peritoneal cells were harvested in phosphate-buffered saline (PBS; 0.01 M, pH 7.2) and centrifuged, and the sediment was resuspended in RPMI 1640 medium supplemented with 10% fetal bovine serum. Cells (1 × 10^5^) were seeded on 13-mm coverslips in 24-well plates and incubated at 37°C in a 5% CO_2_ atmosphere. After 15 h, macrophages were infected with promastigotes at a 10:1 parasite:cell ratio and incubated again for 6 h. The remaining noninternalized parasites were removed. The infected host cells were treated with parthenolide at concentrations of 4.0, 3.2, 2.4, and 1.6 μM. After 24 h, the coverslips were washed with PBS, fixed in methanol, stained with Giemsa, mounted in Entellan (Merck), and examined under an optical microscope. The rate of cell infection and number of amastigotes per cell were evaluated by counting 200 random cells in duplicate cultures in at least two independent experiments. The survival index was calculated by multiplying the percentage of infected macrophages and mean number of internalized parasites per macrophage. Data were compared via one-way analysis of variance (ANOVA) followed by Tukey’s multiple range test for statistically significant differences at p < 0.05.

### Genotoxicity study

To assess the toxicity of parthenolide in mice, a micronucleus test was conducted in groups of five male and five female Swiss albino mice (*Mus musculus*) that weighed approximately 42 g. The animals were obtained from the Central Animal House of the Universidade Estadual de Maringá, Paraná, Brazil. They were housed in plastic cages at 22 ± 1°C and 55 ± 10% humidity, with a 12 h/12 h light/dark cycle and free access to water and food (Nuvilab Cr1). The study was conducted according to experimental standards approved by the Animal Ethics Committee of the Universidade Estadual de Maringá (protocol no. 013/2010).

The animals received 3.75 mg parthenolide/kg body weight suspended in 10% DMSO by oral gavage. The negative control was a vehicle group, and the positive control was a group that received 40 mg cyclophosphamide/kg body weight. The mice were examined regularly for mortality and clinical signs of toxicity until sacrifice by carbon dioxide asphyxiation, which occurred 24 h after treatment. Both femurs were dissected, and bone marrow was flushed with fetal calf serum. After centrifugation for 5 min at 2,000 × *g*, 10 μl of the sediment was smeared on glass slides and air-dried. The smears were fixed with absolute methanol for 5 min and stained with May-Grunwald-Giemsa to detect micronucleated polychromatic erythrocytes (MNPCE). The number of micronucleated cells was counted in 2,000 reticulocytes per animal using an Olympus BH-2 microscope at 1,000× magnification [26]. The statistical analyses were made with a one-way analysis of variance (ANOVA) followed by Dunnet test. Differences were considered significant at p value of less than 0.05.

### Scanning and transmission electron microscopy

After treatment with the IC_50_ (72 h) of parthenolide, axenic amastigotes were washed in PBS and fixed in 2.5% glutaraldehyde in 0.1 M sodium cacodylate buffer at 4ºC. For scanning electron microscopy, amastigotes were placed on a specimen support with a poly-L-lysine-coated coverslip and washed in cacodylate buffer. The cells were dehydrated in an increasing ethanol gradient, critical-point-dried in CO_2_, sputter-coated with gold, and observed in a Shimadzu SS-550 SEM scanning electron microscope.

For transmission electron microscopy, amastigote forms were treated with the IC_50_ of parthenolide and the IC_50_ of amphotericin B and fixed as described above. The cells were postfixed in a solution that contained 1% osmium tetroxide, 0.8% potassium ferrocyanide, and 10 mM calcium chloride in 0.1 M cacodylate buffer, dehydrated in an increasing acetone gradient, and embedded in Epon resin. Ultrathin sections were stained with uranyl acetate and lead citrate, and the images were examined in a Zeiss 900 transmission electron microscope.

### Fluorescence of monodansylcadaverine during cell death

Axenic amastigotes were treated with IC_50_ and IC_90_ equivalents of parthenolide. After 72 h, the cells were washed and resuspended in PBS. To verify the induction of autophagy by parthenolide, the cells were incubated with 0.05 mM monodansylcadaverine (MDC) at 37°C for 10 min. After incubation, the cells were washed three times with PBS to remove excess MDC, immediately analyzed by fluorescence microscopy at an excitation wavelength of 360–380 nm and emission wavelength of 525 nm, and photographed using a charge-coupled-device camera. This study was qualitative.

### Flow cytometry

The antileishmanial activity of parthenolide (20 and 40 μM) on the integrity of the plasma membrane and mitochondrial membrane potential of axenic amastigotes (5 × 10^6^ cells/ml) was determined after 3 h treatment. Amphotericin B (5.0 μM) and carbonyl cyanide *m*-chlorophenylhydrazone (200 μM) were used as positive controls. Untreated amastigotes were used as a negative control. Each flow-cytometric technique was evaluated by repeating each experiment three times to verify reproducibility.

The integrity of the plasma membrane was assessed using *L. amazonensis* amastigotes at an average density of 5 × 10^6^ cells suspended in 500 μl PBS and stained with 50 μl propidium iodide (2 μg/ml) for 5 min at room temperature.

To measure mitochondrial membrane potential (ΔΨ_m_), 1 ml of saline that contained 1 × 10^6^ of treated amastigotes was mixed with 1 μl rhodamine 123 (5 mg/mL) for 15 min at 37°C. The cells were washed, resuspended in PBS, and incubated at the same temperature for 30 min.

A total of 10,000 events were analyzed per sample using a FACSCalibur cytometer, and numeric data were processed with Cellquest software (both from Becton Dickinson). Propidium iodide and rhodamine 123 are excited with a 480 nm argon ion laser, and fluorescence emission occurs at 560–580 nm and 515–530 nm, respectively.

### Electron paramagnetic resonance spectroscopy

Spin-label 5-doxyl stearic acid (5-DSA), with a nitroxide radical moiety (doxyl) in the fifth carbon atom of the acyl chain, was purchased from Sigma (St. Louis, MO, USA). A small aliquot (3 μl) of stock solution of the spin label in ethanol (2 mg/ml) was transferred to a glass tube. After the solvent evaporated, approximately 2.4 × 10^8^ cells of *Leishmania* suspended in 40 μl PBS was added to the film of the spin label with gentle agitation. In a second tube, 6 μl of a stock solution of parthenolide in chloroform (201 mM) was added. After evaporation of the solvent, the first spin-labeled cell suspension was placed on the parthenolide film and gently agitated. The cells were then introduced into a 1 mm inner diameter capillary column for electron paramagnetic resonance (EPR) measurements, which was sealed by flame. Samples were also prepared that contained double and triple the concentrations of parthenolide used in the first sample (using 12 and 18 μl of the solution of parthenolide in chloroform, respectively).

Electron paramagnetic resonance spectroscopy was performed with a Bruker ESP 300 spectrometer (Rheinstetten, Germany) equipped with an ER 4102 ST resonator. The instrument settings were the following: microwave power, 10 mW; modulation frequency, 100 KHz; modulation amplitude, 1.0 G. Electron paramagnetic resonance spectra simulations were performed using the NLLS program developed by Budil and coworkers [48]. In the spectral calculations, the NLLS program includes the magnetic g- and A-tensors and rotational diffusion tensor, *R*, which are expressed in a system of Cartesian axes fixed in the spin-labeled molecule. To reduce the number of parameters in the fittings and simplify the simulation, the average rotational diffusion rate, *R*_*bar*_, was calculated by the fitting program using the relationship *R*_*bar*_ *= (R*_*per*_^*2*^*•R*_*par*_*)*^*1/3*^, in which *R*_*per*_ is the perpendicular component of the rotational diffusion, and *R*_*par*_ is the parallel component of the rotational diffusion. *R*_*bar*_ was converted to the parameter rotational correlation time, *τ*_*c*_, following the relationship *τ*_*c*_ *= 1/6 R*_*bar*_. Similar to previous studies [49,50], the magnetic parameters were determined based on a global analysis of the overall spectra obtained in this work, and all of the EPR spectra were simulated using the same predetermined parameters. In this work, the spectra were simulated with a model of two spectral components.

## Competing interests

The authors declare that they have no competing interests.

## Authors’ contributions

TST conceived and designed the study, carried out all the experimental studies and drafted the manuscript. TUN participated in the design of the study. AA assisted with EPR spectra and helped to draft the manuscript. CVN conceived of the study, and participated in its design and coordination and helped to draft the manuscript. All authors read and approved the final manuscript.
